# A Machine Learning Model for Predicting In-Hospital Mortality in Chinese Patients With ST-Segment Elevation Myocardial Infarction: Findings From the China Myocardial Infarction Registry

**DOI:** 10.2196/50067

**Published:** 2024-07-30

**Authors:** Jingang Yang, Yingxue Li, Xiang Li, Shuiying Tao, Yuan Zhang, Tiange Chen, Guotong Xie, Haiyan Xu, Xiaojin Gao, Yuejin Yang

**Affiliations:** 1 State Key Laboratory of Cardiovascular Disease Fuwai Hospital, National Center for Cardiovascular Diseases Chinese Academy of Medical Sciences & Peking Union Medical College Beijing China; 2 Ping An Healthcare and Technology Beijing China

**Keywords:** ST-elevation myocardial infarction, in-hospital mortality, risk prediction, explainable machine learning, machine learning, acute myocardial infarction, myocardial infarction, mortality, risk, predication model, china, clinical practice, validation, patient management, management

## Abstract

**Background:**

Machine learning (ML) risk prediction models, although much more accurate than traditional statistical methods, are inconvenient to use in clinical practice due to their nontransparency and requirement of a large number of input variables.

**Objective:**

We aimed to develop a precise, explainable, and flexible ML model to predict the risk of in-hospital mortality in patients with ST-segment elevation myocardial infarction (STEMI).

**Methods:**

This study recruited 18,744 patients enrolled in the 2013 China Acute Myocardial Infarction (CAMI) registry and 12,018 patients from the China Patient-Centered Evaluative Assessment of Cardiac Events (PEACE)-Retrospective Acute Myocardial Infarction Study. The Extreme Gradient Boosting (XGBoost) model was derived from 9616 patients in the CAMI registry (2014, 89 variables) with 5-fold cross-validation and validated on both the 9125 patients in the CAMI registry (89 variables) and the independent China PEACE cohort (10 variables). The Shapley Additive Explanations (SHAP) approach was employed to interpret the complex relationships embedded in the proposed model.

**Results:**

In the XGBoost model for predicting all-cause in-hospital mortality, the variables with the top 8 most important scores were age, left ventricular ejection fraction, Killip class, heart rate, creatinine, blood glucose, white blood cell count, and use of angiotensin-converting enzyme inhibitors (ACEIs) and angiotensin II receptor blockers (ARBs). The area under the curve (AUC) on the CAMI validation set was 0.896 (95% CI 0.884-0.909), significantly higher than the previous models. The AUC for the Global Registry of Acute Coronary Events (GRACE) model was 0.809 (95% CI 0.790-0.828), and for the TIMI model, it was 0.782 (95% CI 0.763-0.800). Despite the China PEACE validation set only having 10 available variables, the AUC reached 0.840 (0.829-0.852), showing a substantial improvement to the GRACE (0.762, 95% CI 0.748-0.776) and TIMI (0.789, 95% CI 0.776-0.803) scores. Several novel and nonlinear relationships were discovered between patients’ characteristics and in-hospital mortality, including a U-shape pattern of high-density lipoprotein cholesterol (HDL-C).

**Conclusions:**

The proposed ML risk prediction model was highly accurate in predicting in-hospital mortality. Its flexible and explainable characteristics make the model convenient to use in clinical practice and could help guide patient management.

**Trial Registration:**

ClinicalTrials.gov NCT01874691; https://clinicaltrials.gov/study/NCT01874691

## Introduction

Acute myocardial infarction (AMI) is a major cause of hospitalization and mortality in China, while ST-segment elevation myocardial infarction (STEMI) accounts for over 80% of myocardial infarctions [1-3]. It is critical to accurately predict the risks of in-hospital mortality for patients with STEMI to improve prognosis. Traditionally, most risk prediction models have been based on generalized linear regression methods [4,5]. Although straightforward to understand and apply, these models require parametric assumptions [6,7]. For example, using the logistic regression (LR) method, the Global Registry in Acute Coronary Events (GRACE) [4] and Thrombolysis in Myocardial Infarction (TIMI) risk scores [5] oversimplified the complexity of the real association among variables and outcome, resulting in poor predictive accuracy [8,9]. Recently, machine learning (ML) techniques have been increasingly used for predicting different clinical events in cardiovascular disease [10-12] and have achieved higher accuracy than traditional models. However, ML models, often built on a large number of variables, are difficult to use in clinical practice due to the need for extensive input data and the challenge of identifying specific therapeutic targets. The complexity and ambiguity of ML models require a shift toward explainable artificial intelligence (XAI) methods to guarantee that the model outputs are comprehensible for end users [13]. Moreover, ML models tend to use a large number of variables [14-16]. However, in clinical practice, where many scenarios are unknown, a significant challenge is how to apply the model more flexibly when some variables are missing. Therefore, we aimed to develop an ML risk prediction model for in-hospital mortality in patients with STEMI that is not only highly accurate but also explainable and flexible with the number of input variables (tolerant to the missing variables), making it easy to use in clinical practice.

## Methods

### Data Description

The patients included in this study were from the China Acute Myocardial Infarction (CAMI) registry [3], organized and conducted by the Fuwai Hospital, National Center for Cardiovascular Diseases, China, from January 2013 to September 2014. The methodology of the CAMI registry (NCT01874691) has been previously described [3]. In short, the CAMI registry was a prospective, nationwide, multicenter observational study for patients with AMI. The registry included 3 levels of hospitals (provincial, prefecture, and county), reflecting the typical Chinese governmental and administrative model and providing broad geographic representation across all provinces and municipalities across mainland China. Patients with AMI were consecutively enrolled, and data were collected upon their arrival and throughout their hospital stay until discharge. Data were collected, validated, and submitted by trained clinical cardiologists or cardiovascular fellows to ensure accuracy and reliability at each participating site. Patients diagnosed as non-STEMI (NSTEMI) or lack of in-hospital mortality status were excluded from the study.

The CAMI registry data were used for model derivation and internal validation. Patients with STEMI hospitalized in 2014 (n=9616, 51.3%) were used to derive the model, while those hospitalized in 2013 (n=9125, 48.7%) were used for internal validation. An independent cohort of patients from the China Patient-Centered Evaluative Assessment of Cardiac Events (PEACE)-Retrospective Acute Myocardial Infarction Study [2], another nationally representative sample of patients with STEMI spanning from 2001 to 2011 (N=12,108), was also used to externally validate the proposed risk prediction model. We only selected 10 important variables to carry out the validation, with the aim of assessing the proposed risk prediction model’s flexibility when applied in daily clinical practice. The internal validation set sampled at a different time point, along with the independent external validation set, were both used to assess the model’s reproducibility and generalizability to new and different patients.

### Ethical Considerations

Both study protocols conformed to the ethical guidelines of the 1975 Declaration of Helsinki and were approved by the ethics review board committee of Fuwai Hospital (431) [2,3]. Written informed consent was obtained from eligible patients before registration. All data were anonymized.

### Main Outcome

The main outcome was all-cause in-hospital mortality, defined as death for any reason during hospitalization.

### Predictor Variables

The patients with STEMI included in the CAMI cohort were characterized by a total of 89 variables (Table S1 in Multimedia Appendix 1), including social demographics, presentation characteristics, laboratory tests, treatment, medical history, and more [3]. The patients with STEMI included in the China PEACE-Retrospective Acute Myocardial Infarction Study [2] were characterized by 10 variables, including age, weight, Killip class, heart rate, systolic blood pressure (SBP), glucose, creatinine, white blood cell (WBC) count, high-density lipoprotein cholesterol (HDL-C), and use of angiotensin-converting enzyme inhibitors (ACEIs) or angiotensin II receptor blockers (ARBs).

### Explainable ML Analysis

#### Model Construction

The predictive model was developed using the Extreme Gradient Boosting (XGBoost) [17] approach based on the CAMI derivation set. XGBoost ensembles [18] a series of relatively weak base classifiers (typically decision trees) into a stronger one sequentially and has achieved state-of-the-art results in many clinical challenges [10,19]. Apart from its highly competitive and accurate predictive performance, we chose the XGBoost method for its ability to handle missing data automatically [17]. Users do not need to impute the missing values when deriving, validating, and applying the XGBoost model. XGBoost provides the importance score of each variable, representing the frequency that a variable is used across all trees. The hyperparameters in the XGBoost model were tuned by 5-fold cross-validation on the derivation set.

#### Model Interpretation

The Shapley Additive Explanations (SHAP) method [20] was used to interpret the derived XGBoost model. It offers explanations on how the XGBoost model makes predictions and interprets the complex nonlinear relationship among the predictors and outcomes [19]. This method has been applied recently in other clinical studies [10,19]. SHAP represents the predicted risk as a cumulative effect of contributing variables for each prediction. The variable impact values that SHAP computes essentially represent the change in the predicted risk of the XGBoost model when we observe a feature (such as the weight of a patient) versus when we do not observe the feature (such as not knowing a patient’s weight).

#### Model’s Flexibility in Application

XGBoost’s ability to handle missing values automatically makes it a robust and flexible choice for dealing with input variables. Users are free to input any number of available variables and leave other unrecorded ones as “N/A” (not available) values. Several experiments were conducted to assess the XGBoost model’s flexibility. First, we retained the top 20, 15, and 10 most important variables and replaced the others with “N/A” values on the CAMI derivation set. Second, we randomly reduced the number of available variables from 89 to 10 in the CAMI validation set (Multimedia Appendix 1). Third, we included 10 variables from the independent China PEACE data set for our analysis.

### Statistical Analysis

Descriptive statistics were estimated as mean (SD) for the continuous variables and frequency (percentage) for the categorical ones. The missing rates for each variable were also calculated. Missing values were imputed using the chained equation method proposed in the Multiple Imputation by Chained Equations (MICE) algorithm [21], as the models being compared—namely, lasso LR, random forest, TIMI scores, and GRACE scores—cannot handle missing data automatically. The discrimination ability was estimated by the area under the curve (AUC). Isotonic regression [22] was used downstream of the XGBoost model to adjust the predictions [23,24]. The calibration was assessed using the Hosmer-Lemeshow goodness-of-fit test [25] on the CAMI derivation set. Additionally, a decile plot of observed versus predicted risk was used to visualize the calibration.

## Results

### Overview

The in-hospital mortality rate was 6.9% (n=663), 6.8% (n=621), and 9.3% (n=1132) in the CAMI derivation, validation, and China PEACE sets, respectively. The descriptive statistics of the CAMI and China PEACE data set are illustrated in Table S2 in Multimedia Appendix 1, while the missing rates are listed in Table S3 in Multimedia Appendix 1.

### Prediction of In-Hospital Mortality

**Figure 1:**
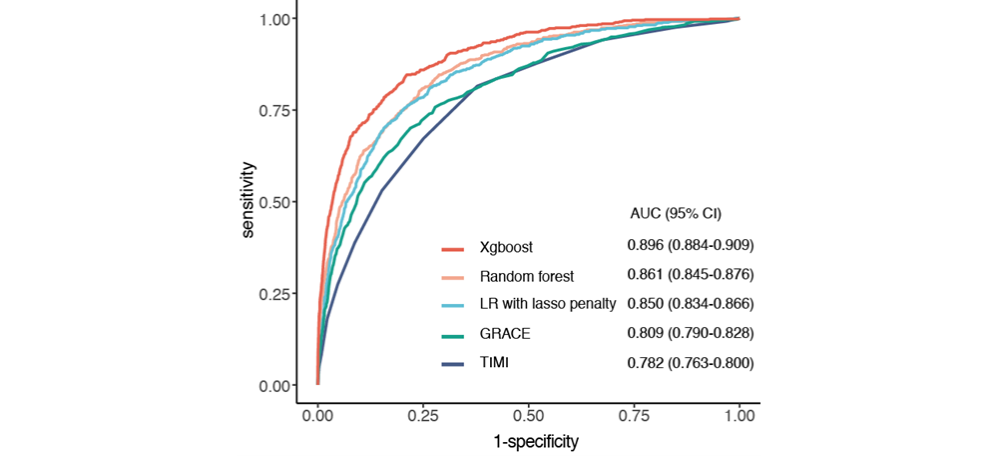
Receiver operating characteristic (ROC) curve of different compared models on the China Acute Myocardial Infarction (CAMI) validation set. GRACE: Global Registry in Acute Coronary Events; LR: logistic regression; TIMI: thrombolysis in myocardial infarction; XGBoost: Extreme Gradient Boosting.

Figure 1 illustrates the receiver operating characteristic (ROC) curves of all the compared models. XGBoost produced the highest discrimination performance for in-hospital mortality with an AUC of 0.896 (95% CI 0.884-0.909; *P*<.05) on CAMI validation set, better than the 2 compared ML methods: random forest (AUC 0.861, 95% CI 0.845-0.876) and LR with lasso penalty (0.850, 95% CI 0.834-0.866). The XGBoost model also exhibited a significant improvement over the 2 well-established models: GRACE score (AUC 0.809, 95% CI 0.790-0.828) and TIMI score (AUC 0.782, 95% CI 0.763-0.800)*.* All comparisons were statistically significant when *P*<.05.

**Figure 2:**
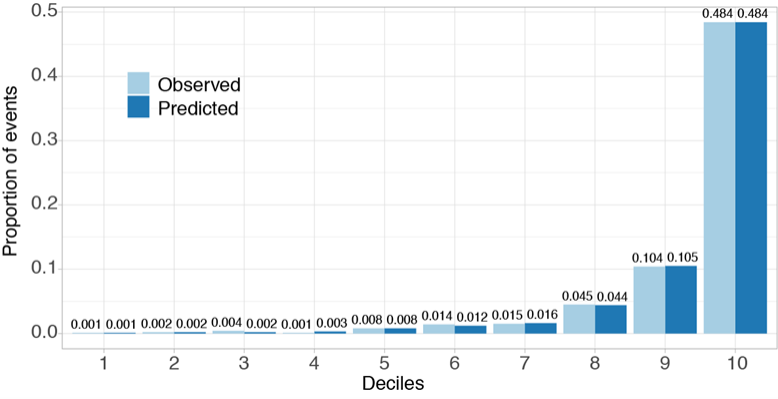
Calibration plot for the Extreme Gradient Boosting (XGBoost) model. The calibration plot shows the relationship between the observed and predicted in-hospital mortality, grouped by deciles of predicted risk. The XGBoost model showed excellent calibration with the observed in-hospital mortality.

The Hosmer-Lemeshow statistic for the XGBoost model was 2.378 (*P*=.97), indicating a very good calibration result. The decile plot further confirmed strong agreement between XGBoost predicted probability and the observed in-hospital mortality risk (Figure 2).

The hyperparameters for XGBoost and random forest, tuned by 5-fold cross-validation, are listed in Tables S4 and S5 in Multimedia Appendix 1.

### Model Interpretation

**Figure 3:**
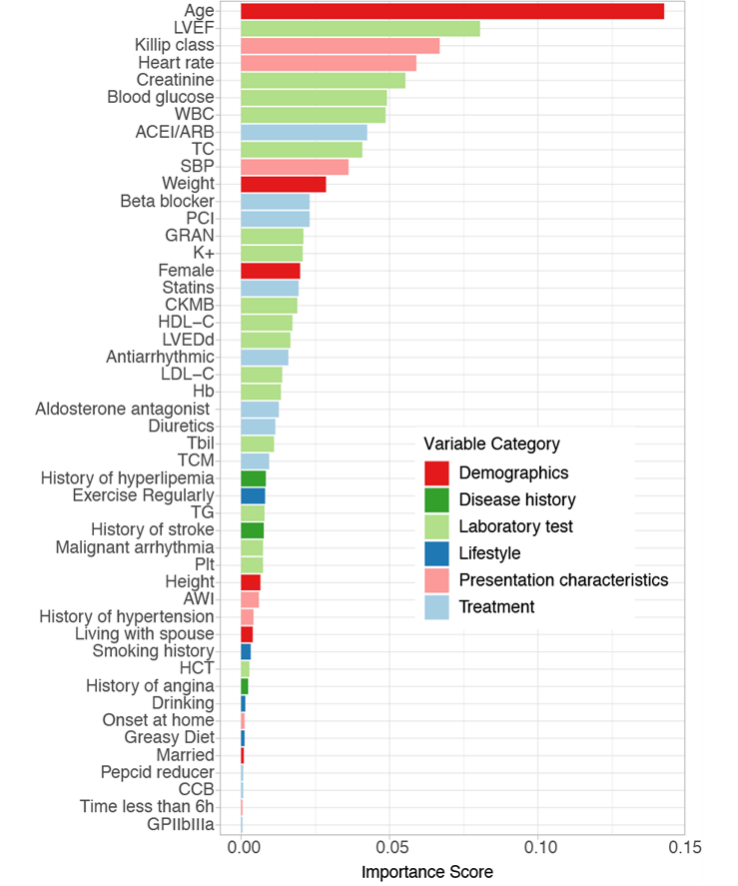
Variable importance score in the Extreme Gradient Boosting (XGBoost) model. A total of 48 variables with importance scores over 0 are illustrated. The color of the bar represents the variable category. ACEI: angiotensin-converting enzyme inhibitor; ARB: angiotensin II receptor blocker; AWI: anterior wall infarction; CCB: calcium channel blocker; CKMB: creatine kinase Mb isoenzyme; GRAN: neutrophilic granulocyte; HCT: Hematocrit; HDL-C: high-density lipoprotein-cholesterol; LDL-C: low-density lipoprotein-cholesterol; LVEDd: left ventricular end diastolic diameter; LVEF: left ventricular ejection fraction; PCI: percutaneous coronary intervention; SBP: systolic blood pressure; TC: total cholesterol; TCM: traditional Chinese medicine; TG: triglyceride; WBC: white blood cell.

Figure 3 illustrates the variable importance score in the XGBoost model, reflecting the frequency with which a variable was used across all trees. Age was the most important predictor of in-hospital mortality, followed by left ventricular ejection fraction (LVEF), Killip class, heart rate, creatinine, and blood glucose.

**Figure 4:**
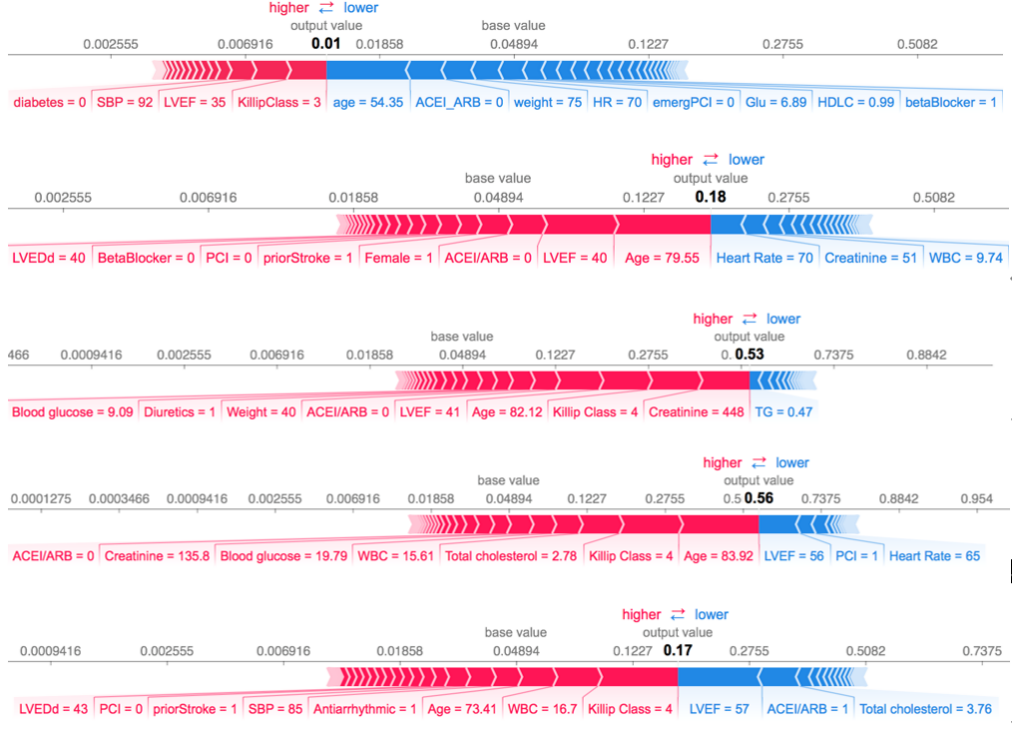
Sample of predicted risk of in-hospital mortality for a selected patient. ACEI: angiotensin-converting enzyme inhibitor; ARB: angiotensin II receptor blocker; HR: heart rate; Glu: glucose, LVEF: left ventricular ejection fraction; PCI: percutaneous coronary intervention; SBP: systolic blood pressure; WBC: white blood cell.

Figure 4 explains the rationale behind the model’s prediction of an individual’s risk. It displays the relative contributions of all features toward the predicted risk of in-hospital mortality. For instance, a predicted risk value of 0.01 for illustrated patient A was influenced by variables such as Killip class, LVEF, age, weight, and use of ACEI/ARB, among others. The red bars in Figure 3 indicate variables that increase the risk (pushing to the right), while the blue bars indicate variables that decrease the risk (pushing to the left). The length of each bar corresponds to the magnitude of its effect.

**Figure 5:**
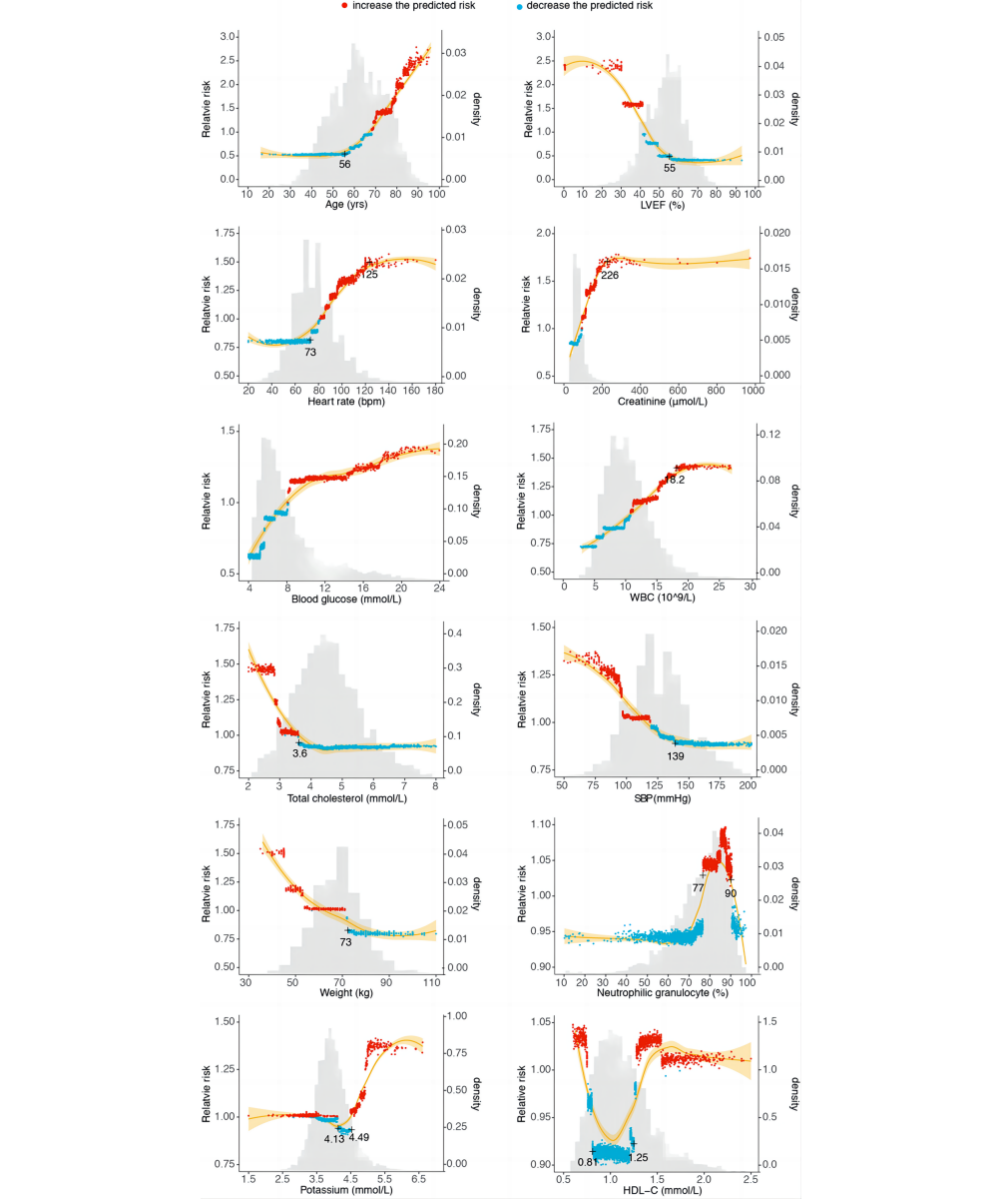
Effect of varying individual variable values on the in-hospital mortality. These partial dependence plots show the change in in-hospital mortality risk for all values of a given feature. The grey histograms on each plot show the distribution of values for that variable in the derivation data set. Each point in red was obtained from one prediction. The green lines were fitted from the points by smoothed conditional means. HDL-C: high-density lipoprotein cholesterol; LVEF: left ventricular ejection fraction; SBP: systolic blood pressure; WBC: white blood cell.

Figure 5 shows important novel and nonlinear relationships between individual variables and in-hospital mortality risk captured by the XGBoost model. For example, when age was less than 56 years, its attribution to in-hospital mortality was consistent and increased linearly after age was higher than 56 (J-shaped relationship). The heart rate variable displayed an S-shaped relationship with in-hospital mortality risk. The risk increased linearly after the heart rate was higher than 73 bpm and almost doubled until it reached 125 bpm. LVEF followed an inverted S-shaped pattern. Creatinine increased linearly until 26 and became constant after that (inverted J-shaped relationship), similar to WBC. Higher blood glucose reflected an increased in-hospital mortality risk. Variables like total cholesterol, SBP, and weight showed an L-shaped pattern. An N-shaped relationship was shown for neutrophilic granulocytes. Patients with neutrophilic granulocytes between 77% and 90% were predicted to have a higher in-hospital mortality risk. HDL-C displayed a U-shaped pattern. For potassium, a value between 4.13 and 4.49 mmol/L predicted the lowest in-hospital mortality risk.

### Flexibility of the Predictive Model

When we retained the top 20, 15, and 10 most important variables (Figure 2) and replaced others as “N/A” values in the CAMI validation set, the XGBoost model still achieved an AUC of 0.892 (95% CI 0.879-0.905), 0.885(95% CI 0.872-0.899), and 0.877(95% CI: 0.862 0.891), respectively. When the number of retained variables was reduced randomly from 89 to 10, the AUC decreased from 0.896 to 0.825 (SD 0.020) (20 available variables) to 0.810 (SD 0.011) (10 available variables) (Figure S1 in Multimedia Appendix 1). When the XGBoost model was validated on the China PEACE data set with the top 10 available variables (Figure 2), it achieved an AUC of 0.840 (95% CI 0.829-0.852). For comparison, the TIMI score and GRACE score applied to the China PEACE data set gained AUCs of 0.762 (95% CI 0.748-0.776) and 0.789 (95% CI 0.776-0.803). The XGBoost model still significantly outperformed the conventional TIMI and GRACE risk score models.

For practical convenience, we embedded the XGBoost prediction model in a web-based calculator that required only the top 10 most important variables as inputs [19].

## Discussion

In this study, we proposed a risk model that predicted in-hospital mortality for patients with STEMI by incorporating the ML method XGBoost and the model interpretation approach SHAP. The model we constructed had excellent performance in terms of high predictive accuracy, high tolerance to missing values (flexibility), and good clinical interpretability. Importantly, we identified the top 7 clinical factors affecting in-hospital mortality as age, LVEF, Killip class, heart rate, creatinine, glucose, and WBC. Among these, LVEF, glucose, and WBC were not included in the current traditional predictive models. Although creatinine is also included in the GRACE score, its relationship with mortality is not a simple linear one. The predictive value of glucose and WBC exceeds that of other variables in traditional predictive models, such as blood pressure, weight, and medical history (hypertension, diabetes, and angina). We believe that these findings can help doctors understand the value of ML models and uncover the pathophysiological significance of certain clinical variables in myocardial infarction.

While traditional statistical models such as TIMI and GRACE, as recommended by current guidelines [26], are useful and user-friendly, their overly simplified nature may result in inadequate predictive accuracy for risk classification and decision-making [8]. First, these models are developed based on a limited number of variables and may not encompass comprehensive information. Second, the LR method used by these models requires strong assumptions, including a linear relationship under the logit function, independence of observations, and no multicollinearity among variables [7,8,25,27]. This results in underestimating the complexity of the real association among variables and outcomes.

In contrast, ML methods can handle a larger number of variables, require no parametric assumptions, and can learn the complex relationships hidden in the data automatically [9]. The XGBoost method overcomes these limitations by generating a series of classification and regression trees (CARTs) with each one learning the residuals of its predecessors. The boosting mechanism gives the model a strong predictive power. As observed, the XGBoost model achieved an impressive AUC of 0.896 (95% CI 0.884-0.909) on the CAMI validation set, outperforming the other methods and proving to be a more powerful and effective tool for clinical risk prediction.

The XGBoost model’s ability to tolerate missing values makes it well-suited for clinical applications, where incomplete variables are frequent [28-30]. While most ML methods achieve accuracy and precision by learning from a large number of variables, they often lose practicality because it is usually difficult to collect all the predictors used in the model in clinical practice. In such cases, missing values must be imputed if clinicians still want to apply the model. The proposed XGBoost model overcomes this weakness thanks to its ability to deal with missing values. We demonstrated that the XGBoost model’s performance is relatively robust when faced with incomplete data compared to the traditional LR model. Even with only the top 10 important variables, the XGBoost model achieved an AUC of 0.877(95% CI 0.862-0.891) on the CAMI validation set. On the independent China PEACE set with only the top 10 important variables available, XGBoost gained an AUC of 0.840 (95% CI 0.829-0.852) compared to TIMI 0.762 (95% CI 0.748-0.776) and GRACE 0.789 (95% CI 0.776-0.803). These results demonstrated the XGBoost model’s flexibility and generalization ability, which could alleviate concerns about the feasibility of applying complex ML models in clinical practice.

Another concern about the complex ML approaches applied in clinical practice is their lack of transparency. Unlike the widely employed LR method, whose coefficients clearly indicate the effect of predictive factors on the outcome, the black-box nature of complex ML algorithms applied in medical tasks has been seriously criticized and doubted in recent years [8,9]. To address this issue, our study used SHAP to interpret how the predicted risk was determined for individual patients and uncover the complex relationship between predictors and outcomes embedded in the XGBoost model.

Our results showed that HDL-C displayed a U-shaped relationship with in-hospital mortality among patients with STEMI. In the previous studies, Madsen et al [31] reported a U-shaped association between HDL-C and mortality, using data from 52,268 men and 64,240 women enrolled in 2 prospective population-based studies. Similarly, Bowe et al [32] found a U-shaped relationship between HDL-C and the risk of all-cause mortality in patients with kidney disease. For the variable potassium, our result showed that the patients with STEMI with potassium levels ranging from 4.13 to 4.49 mmol/L had the lowest in-hospital mortality risk, while levels greater than 4.5 mmol/L increased the mortality risk. Clinical practice guidelines recommend maintaining serum potassium levels between 4.0 and 5.0 mmol/L in patients with acute myocardial infarction (AMI) [33,34]. However, recent studies have challenged these guidelines, reporting that potassium levels greater than 4.5 mmol/L are associated with increased mortality [35-37]. Our study found that creatinine >1.1mg/dl (94.5/L) contributed to a higher in-hospital mortality risk. A previous study [38] reported that an elevated serum creatinine level (defined as creatinine ≥1.2 mg/dl) predicted a higher long-term mortality risk in patients with AMI.

For the variable blood glucose, our results showed that levels less than 8.15 mmol/L were safer for patients with STEMI. Another study reported that the best cutoff values for 30-day mortality among patients with STEMI were 149 mg/dL (8.27 mmol/L) for those without diabetes, 231 mg/dL (12.82 mmol/L) for those with diabetes, and 169 mg/dL (9.38 mmol/L) for all patients [39]. For the variable WBC, our result showed that a higher WBC count was associated with higher in-hospital mortality risk, with a safer threshold being less than 10.77/L. Cannon et al [40] reported that mortality at 30 days showed a curvilinear increase with increasing WBC count, with mortality rising in patients with WBC count >10,000 /dL *(P<*.0001). Previous studies often investigated this relationship by categorizing or binning continuous variables and regressing the outcome on the categorical variables. However, this approach is heavily influenced by predefined cutoffs and cannot provide a continuous picture of the relationship. In contrast, our model offered more thorough and quantitative insights into the exact change in risk induced by specific patient characteristics. By interpreting how each variable contributed to in-hospital mortality, our study could help clinicians identify specific therapeutic targets and further guide patient management.

Our research has a certain guiding significance for clinical implementation. First, the new model is significantly superior to traditional GRACE and TIMI models, helping doctors predict patient prognosis. Second, ML has identified several variables not included in past models, which may serve as potential targets for clinical intervention or provide further understanding of the pathophysiology of disease development, such as WBC and blood glucose. Third, while clinicians often find it difficult to understand the variables selected by ML, adopting the XGBoost model and model interpretation approach SHAP further increases accuracy by capturing nonlinear relationships among the predictors and outcomes. This offers a clear explanation for why ML can improve predictive efficiency, thus enhancing clinicians’ understanding of the performance improvement of ML. Methodologically, we used internal validation and a large sample size of independent external validation, all leading to consistent conclusions.

However, despite the superior performance of the proposed XGBoost model, several limitations still exist. First, the proposed XGBoost model was derived and validated on the Chinese STEMI patient cohort. Further validation is needed to confirm its efficiency on more general cohorts. Second, the study was designed prospectively, but this research is a retrospective analysis, so the variables recruited in our study may be limited. The model may be more powerful if more informative variables were added.

In conclusion, the proposed ML model in our paper demonstrated strong advantages in predictive ability, flexibility, and interpretability. Although some results need further study and verification, we have shown the benefits of complex models in the field of disease predictions. We offered a web calculator for convenient application, and we hope our study can help augment and extend the effectiveness of cardiologists to improve patient care and promote incorporating ML into daily practice.

## Appendix

Multimedia Appendix 1 Additional information and tables.

## Abbreviations

ACEI: angiotensin-converting enzyme inhibitor
AUC: area under the curve
ARB: angiotensin II receptor blocker
AMI: acute myocardial infarction
CAMI: China Acute Myocardial Infarction
CART: classification and regression tree
GRACE: Global Registry in Acute Coronary Events
HDL-C: high-density lipoprotein cholesterol
LVEF: left ventricular ejection fraction
LR: logistic regression
MICE: Multiple Imputation by Chained Equations
ML: machine learning
NSTEMI: non–ST-segment elevation myocardial infarction
PEACE: Patient-Centered Evaluative Assessment of Cardiac Events
ROC: receiver operating characteristic
SBP: systolic blood pressure
SHAP: Shapley Additive Explanations
STEMI: ST-segment elevation myocardial infarction
TIMI: Thrombolysis In Myocardial Infarction
WBC: white blood cell
XGBoost: Extreme Gradient Boosting
XAI: explainable artificial intelligence
