# Supplementary material for: A Machine Learning Model for Predicting In-Hospital Mortality in Chinese Patients With ST-Segment Elevation Myocardial Infarction: Findings From the China Myocardial Infarction Registry
Source: J Med Internet Res. 2024 Jul 30;26:e50067. doi: 10.2196/50067 (PMC11322712; doi:10.2196/50067)
Supplement: Multimedia Appendix 1 [file jmir_v26i1e50067_app1.docx]

Supplementary material

### Model flexibility (Tolerance to missing values)

To further examine the flexibility of the proposed XGBoost model, we randomly replaced the original variables with *NA* values in the CAMI validation set. During the procedure, four easily obtained variables (age, Killip, heart rate and systolic blood pressure) were fixed. Then we calculated the AUCs with the XGBoost model which was built on the 89 variables. The number of the missing variables varied from 0 to 79 and each was repeated 100 times. Mean and standard deviation of the 100 AUCs were calculated. For comparison, we also measured the flexibility of the LR model with 89 predictors derived on the CAMI derivation set. As it couldn’t deal with the missing variables automatically, we filled the missing variables with mean values after replacing them with *NA* values.

XGBoost gained higher AUC than LR while the number of missing variables increased from 0 to 79. The AUCs were 0.896 with full 89 variables, $0.825\pm0.020$with 20 available variables (69 missing variables) and $0.810\pm0.011$with 10 available variables (79 missing variables) in the XGBoost model while 0.860, $0.753\pm0.035$and $0.722\pm0.028$respectively in the LR model. The result indicated that the XGBoost model was feasible in the clinical practice

**Figure S1. AUCs on CAMI validation data with different number of missing variables**. The x-axis was the number of missing variables and the y-axis was the AUC score. The red line belonged to the XGBoost model while the blue line belonged to the LR model. Points denoted the mean AUC of the 100 rounds of calculation. The error bar denoted the standard deviation. The XGBoost and LR model were both constructed based on 89 variables.


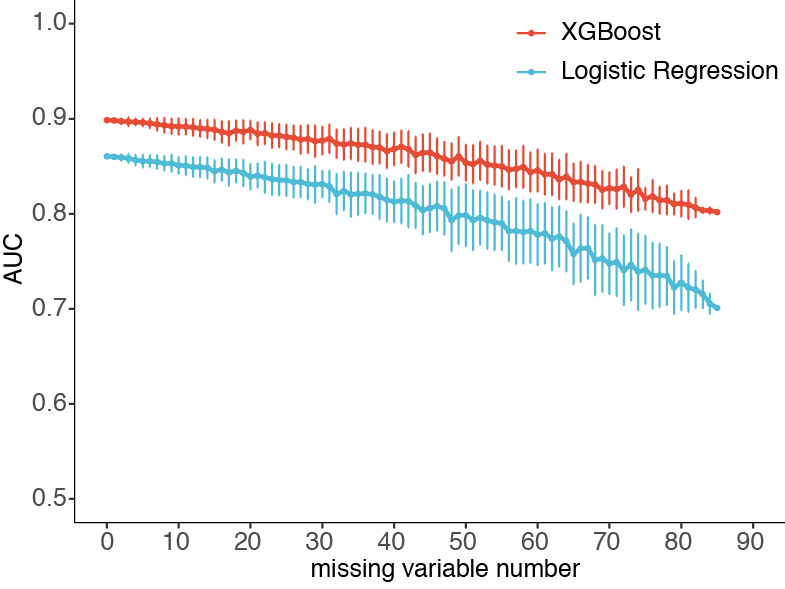


**Table S1. Eighty-nine variables in CAMI validation set**

| Demographics | Age (yrs), Female, Height (cm), Weight (kg), Ethnicity-Han, Married, Education level-illiteracy, Unemployment, Living with spouse, Hospital level (County, City, Province) |
| --- | --- |
| Presentation characteristics | Killip (I, II, III, IV), Heart Rate (bpm), SBP^a^ (mmHg), Times of angina within 24hrs, Time from symptom to hospital (<3h, 3-6h, 6-12h, 12-24h, 1-7day), OnsetAtHome, Persistent chest pain, Atypical Presentations, EMS^b^, AWI^c^, Predisposing factor, Prodromus, Cardiac arrest, Hypertension |
| Laboratory test | Glucose (mmol/L), Creatinine (μmol/L), Hemoglobin (g/L), Hematocrit, Platelet count(×10^9^/L), WBC^d^ (×10^9^/L), GRAN^e^ (%), TC^f^ (mmol/L), L-DLC^g^ (mmol/L), H-DLC^h^ (mmol/L), TG^i^ (mmol/L), K+^g^ (mmol/L), Serum bilirubin (μmol/L), LVEDd^k^(mm), LVEF^l^ (%), Malignant arrhythmia, CKMB^m^(IU) |
| Treatment | Primary PCI^n^, Emergency CABG^o^, Thrombolysis, Aspirin, P2Y12, GPIIbIIIa, Heparin, Oral anticoagulant, Statins, Nitrates, Betablocker, Calcium channel blocker, ACEI/ARB, Antiarrhythmic drug, Aldosterone antagonist, Diuretics, Non-statin lipid-lowering drug, Acid-suppressing, TCM^p^ |
| Medical History | History of smoking , History of drinking, Dyslipidemia, Diabetes, Prior Angina, Prior MI, Prior CABG, Prior Heart Failure, Prior stroke, Prior PVD, Prior aortic disease, Chronic Kidney Disease, COPD^q^, History of Rheumatic diseases, History of cancer, History of stomach ulcer, History of liver disease, History of major bleeding |
| Living habit | Current drinking, Like-greasy, Regular-Exersice, Current smoking |
| Social economics | Hospital-eastern, Hospital equipped with CCU^r^, Hospital-capability of primary PCI, Hospital-northern, Hospital-capability of thrombolysis, Hospitals annually admitting ≥100 patients with AMI^s^, Insurance (Self-paying) |

^a^ **SBP:** systolic blood pressure; ^b^**EMS:** emergency medical service; ^c^**AWI:** anterior wall infarction; ^d^**WBC:** white blood cell; ^e^**GRAN:** neutrophilic granulocyte; ^f^**TC:** total cholesterol; ^g^**LDL-C:** low density lipoprotein-cholesterol; ^h^**HDL-C:** high density lipoprotein-cholesterol; ^i^**TG:** Triglyceride; ^j^**K+:** potassium; ^k^**LVEDd:** left ventricular end diastolic diameter; ^l^**LVEF:** left ventricular ejection fraction; ^m^**CKMB:** creative kinase MB; ^n^**PCI:** percutaneous coronary intervention; ^o^**CABG:** coronary artery bypass graft; ^p^**TCM:** traditional Chinese medicine; ^q^**COPD:** chronic obstructive pulmonary disease; ^r^**CCU:** coronary care unit; ^s^**AMI:** acute myocardial infarction;

##### Table S2. Baseline characteristics

| **Variables** | | **CAMI derivation set (n=9616)** | | **CAMI validation set (n=9125)** | **China PEACE validation set (n=5849)** |
| --- | --- | --- | --- | --- | --- |
| **Demographics** | |  | |  |  |
| Age (yrs), mean(sd) | | 62.10(12.58) | | 61.93(12.34) | 64.83(12.55) |
| Female, n(%) | | 2260(23.50) | | 2169(23.77) |  |
| Height (cm), mean(sd) | | 167.96(7.04) | | 167.60(7.27) |  |
| Weight (kg), mean(sd) | | 67.99(10.56) | | 67.98(10.85) | 66.82(11.94) |
| Ethnicity-Han, n(%) | | 9094(94.54) | | 8613(94.39) |  |
| Married, n(%) | | 8937(92.91) | | 8326(91.24) |  |
| Education level-illiteracy, n(%) | | 779(8.10) | | 659(7.22) |  |
| Unemployment, n(%) | | 698(7.26) | | 584(6.40) |  |
| Living with spouse, n(%) | | 7890(82.03) | | 7346(80.50) |  |
| Hospital level, n(%) | County | 1213(12.61) | | 1369(15) |  |
|  | City | 5185(53.90) | | 4440(48.66) |  |
|  | Province | 3221(33.49) | | 3316(36.34) |  |
| **Presentation characteristics** | | Train | | Test | China PEACE |
| Killip  n(%) | I | 7379(76.71) | | 6738(73.84) | 5823(73.47) |
|  | II | 1469(15.27) | | 1552(17.01) | 963(12.15) |
|  | III | 373(3.88) | | 394(4.32) | 533(6.72) |
|  | IV | 360(3.74) | | 355(3.89) | 400(5.05) |
| Heart Rate (bpm), mean(sd) | | 77.57(18.08) | | 77.59(18.38) | 79.52(19.34) |
| SBP^a^ (mmHg), mean(sd) | | 126.25(24.17) | | 127.19(25.19) | 132.03(25.54) |
| Times of angina within 24hrs, mean(sd) | | 1.25(1.62) | | 1.20(1.74) |  |
| Time from symptom to hospital, n(%) | <3h | 2272(23.6) | | 2211(24.2) |  |
|  | 3-6h | 2599(27.0) | | 2341(25.7) |  |
|  | 6-12h | 1596(16.6) | | 1483(16.3) |  |
|  | 12-24h | 931(9.7) | | 985(10.8) |  |
|  | 1-7day | 2125(22.1) | | 1993(21.8) |  |
| OnsetAtHome, n(%) | | 8496(88.33) | | 7784(85.30) |  |
| Persistent chest pain, n(%) | | 8402(87.35) | | 7888(86.44) |  |
| Atypical Presentations, n(%) | | 380(3.95) | | 414(4.54) |  |
| EMS^b^, n(%) | | 934(9.71) | | 1081(11.85) |  |
| AWI^c^, n(%) | | 5064(52.65) | | 4832(52.95) |  |
| Predisposing factor, n(%) | | 1795(18.66) | | 1694(18.56) |  |
| Prodromus, n(%) | | 2504(26.03) | | 2709(29.69) |  |
| Cardiac arrest, n(%) | | 106(1.10) | | 145(1.59) |  |
| Hypertension, n(%) | | 4442(46.18) | | 4472(49.01) |  |
| **Laboratory test** | |  | |  |  |
| Glucose (mmol/L), mean(sd) | | 8.06(3.45) | | 8.04(3.43) | 8.47(10.28) |
| Creatinine (μmol/L), mean(sd) | | 82.22(44.23) | | 82.18(42.94) | 91.70(72.48) |
| Hemoglobin (g/L), mean(sd) | | 137.67(19.29) | | 137.39(19.39) |  |
| Hematocrit, mean(sd) | | 40.91(5.90) | | 40.70(5.69) |  |
| Platelet count(×10^9^/L), mean(sd) | | 213.76(65.79) | | 210.51(67.15) |  |
| WBC^d^ (×10^9^/L), mean(sd) | | 10.45(3.56) | | 10.48(3.62) | 9.82(18.37) |
| GRAN^e^ (%), mean(sd) | | 76.11(11.92) | | 75.75(12.41) |  |
| TC^f^ (mmol/L), mean(sd) | | 4.55(1.04) | | 4.57(1.07) |  |
| LDL-C^g^ (mmol/L), mean(sd) | | 2.80(0.89) | | 2.84(0.92) |  |
| HDL-C^h^ (mmol/L), mean(sd) | | 1.13(0.31) | | 1.14(0.33) | 1.14(0.35) |
| TG^i^ (mmol/L), mean(sd) | | 1.73(1.25) | | 1.74(1.33) |  |
| K+^j^ (mmol/L), mean(sd) | | 3.96(0.51) | | 3.95(0.52) |  |
| Serum bilirubin (μmol/L), mean(sd) | | 15.21(6.63) | | 15.12(6.61) |  |
| LVEDd^k^(mm), mean(sd) | | 49.30(8.38) | | 49.84(7.19) |  |
| LVEF^l^ (%), mean(sd) | | 53(10.48) | | 52.88(10.69) |  |
| Malignant arrhythmia, n(%) | | 658(6.84) | | 752(8.24) |  |
| CKMB^m^(IU/L), mean(sd) | | 75.82(111.73) | | 71.53(108.75) |  |
| **Treatment, n(%)** | |  | |  |  |
| Primary PCI^n^ | | 4117(42.80) | | 3921(42.97) |  |
| Emergency CABG^o^ | | 17(0.18) | | 11(0.12) |  |
| Thrombolysis | | 894(9.29) | | 953(10.44) |  |
| Aspirin | | 9264(96.31) | | 8699(95.33) |  |
| P2Y12 | | 9263(96.30) | | 8659(94.89) |  |
| GPIIbIIIa | | 3180(33.06) | | 3136(34.37) |  |
| Heparin | | 8500(88.37) | | 8021(87.90) |  |
| Oral anticoagulant | | 189(1.96) | | 83(0.91) |  |
| Statin | | 8702(90.47) | | 8173(89.57) |  |
| Nitrate | | 6679(69.44) | | 6307(69.12) |  |
| Betablocker | | 6542(68.01) | | 6115(67.01) |  |
| Calcium channel blocker | | 1267(13.17) | | 1053(11.54) |  |
| ACEI/ARB | | 5251(54.59) | | 5290(57.97) | 4993(100) |
| Antiarrhythmic drug | | 1063(11.05) | | 910(9.97) |  |
| Aldosterone antagonist | | 2613(27.16) | | 2441(26.75) |  |
| Diuretics | | 3215(33.42) | | 3085(33.81) |  |
| Non-statin lipid-lowering drug | | 162(1.68) | | 161(1.76) |  |
| Acid-suppressing drug | | 6578(68.39) | | 6406(70.20) |  |
| TCM^p^ | | 1625(16.89) | | 1321(14.48) |  |
| **Medical History, n(%)** | |  | |  |  |
| History of smoking | | 5337(55.48) | | 5235(57.37) |  |
| History of drinking | | 4895(50.89) | | 4694(51.44) |  |
| Dyslipidemia | | 506(5.26) | | 673(7.38) |  |
| Diabetes | | 1645(17.10) | | 1648(18.06) |  |
| Prior Angina | | 1947(20.24) | | 2225(24.38) |  |
| Prior MI | | 515(5.35) | | 563(6.17) |  |
| Prior CABG | | 15(0.16) | | 30(0.33) |  |
| Prior Heart Failure | | 113(1.17) | | 143(1.57) |  |
| Prior stroke | | 822(8.55) | | 820(8.99) |  |
| Prior PVD | | 35(0.36) | | 42(0.46) |  |
| Prior aortic disease | | 15(0.16) | | 13(0.14) |  |
| Chronic Kidney Disease | | 65(0.68) | | 80(0.88) |  |
| COPD^q^ | | 151(1.57) | | 167(1.83) |  |
| History of Rheumatic diseases | | 85(0.88) | | 69(0.76) |  |
| History of cancer | | 110(1.14) | | 114(1.25) |  |
| History of stomach ulcer | | 227(2.36) | | 312(3.42) |  |
| History of liver disease | | 91(0.95) | | 97(1.06) |  |
| History of major bleeding | | 142(1.48) | | 176(1.93) |  |
| **Living habit, n(%)** | |  | |  |  |
| Current drinking | | 953(9.91) | | 1094(11.99) |  |
| Like-greasy | | 3140(32.64) | | 2546(27.90) |  |
| Regular-Exersice | | 2212(23) | | 1861(20.39) |  |
| Current smoking | | 4411(45.86) | | 4352(47.69) |  |
| **Social economics, n(%)** | | |  |  |  |
| Hospital-eastern | | | 5080(52.81) | 5116(56.07) |  |
| Hospital equiped with CCU^r^ | | | 9220(95.85) | 8831(96.78) |  |
| Hospital-capability of primary PCI | | | 8988(93.44) | 8389(91.93) |  |
| Hospital-northern | | | 5824(60.55) | 5796(63.52) |  |
| Hospital-capability of thrombolysis | | | 9291(96.59) | 8776(96.18) |  |
| Hospitals annually admitting ≥100 patients with AMI^s^ | | | 7715(80.21) | 7220(79.12) |  |
| Insurance(Self-paying) | | | 718(7.46) | 719(7.88) |  |

^a^ **SBP:** systolic blood pressure; ^b^**EMS:** emergency medical service; ^c^**AWI:** anterior wall infarction; ^d^**WBC:** white blood cell; ^e^**GRAN:** neutrophilic granulocyte; ^f^**TC:** total cholesterol; ^g^**LDL-C:** low density lipoprotein-cholesterol; ^h^**HDL-C:** high density lipoprotein-cholesterol; ^i^**TG:** Triglyceride; ^j^**K+:** potassium; ^k^**LVEDd:** left ventricular end diastolic diameter; ^l^**LVEF:** left ventricular ejection fraction; ^m^**CKMB:** creative kinase MB; ^n^**PCI:** percutaneous coronary intervention; ^o^**CABG:** coronary artery bypass graft; ^p^**TCM:** traditional Chinese medicine; ^q^**COPD:** chronic obstructive pulmonary disease; ^r^**CCU:** coronary care unit; ^s^**AMI:** acute myocardial infarction;

##### Table S3 Missing rate (%) of variables

| **Variables** | **CAMI derivation set (n=9616)** | | **CAMI validation set (n=9125)** | **China PEACE validation set (n=5849)** |
| --- | --- | --- | --- | --- |
| **Demographics** | |  |  |  |
| Age (yrs), mean(sd) | 1.58 | | 1.78 | 0 |
| Female, n(%) | 0 | | 0 | 100 |
| Height (cm), mean(sd) | 3.05 | | 4.62 | 100 |
| Weight (kg), mean(sd) | 3.12 | | 4.65 | 63.17 |
| Ethnicity-Han, n(%) | 1.34 | | 1.1 | 100 |
| Married, n(%) | 1.91 | | 2.48 | 100 |
| Education level-illiteracy, n(%) | 28.64 | | 30.3 | 100 |
| Unemployment, n(%) | 12.63 | | 13.53 | 100 |
| Living with spouse, n(%) | 0.26 | | 0.47 | 100 |
| Hospital level, n(%) | County | 0 | 100 | 100 |
|  | City |  |  |  |
|  | Province |  |  |  |
| **Presentation characteristics** |  |  |  | |
| Killip  n(%) | I | 0.49 | 0.94 | 0 |
|  | II |  |  |  |
|  | III |  |  |  |
|  | IV |  |  |  |
| Heart Rate (bpm), mean(sd) | 0.49 | | 1.13 | 0 |
| SBP^a^ (mmHg), mean(sd) | 1.25 | | 1.75 | 0 |
| Times of angina within 24hrs, mean(sd) | 8.37 | | 11.16 | 100 |
| Time from symptom to hospital, n(%) | <3h | 1 | 1.23 | 100 |
|  | 3-6h |  |  |  |
|  | 6-12h |  |  |  |
|  | 12-24h |  |  |  |
|  | 1-7day |  |  |  |
| OnsetAtHome, n(%) | 0.25 | | 0.41 | 100 |
| Persistent chest pain, n(%) | 1.48 | | 1.82 | 100 |
| Atypical Presentations, n(%) | 1.81 | | 1.87 | 100 |
| EMS^b^, n(%) | 0.34 | | 0.56 | 100 |
| AWI^c^, n(%) | 0.37 | | 0.25 | 100 |
| Predisposing factor, n(%) | 8.75 | | 11.01 | 100 |
| Prodromus, n(%) | 6.1 | | 8.25 | 100 |
| Cardiac arrest, n(%) | 0.37 | | 0.79 | 100 |
| Hypertension, n(%) | 1.81 | | 2.41 | 100 |
| **Laboratory test** |  | |  |  |
| Glucose (mmol/L), mean(sd) | 6.19 | | 5.46 | 11.18 |
| Creatinine (μmol/L), mean(sd) | 4.38 | | 3.91 | 12.94 |
| Hemoglobin (g/L), mean(sd) | 3.93 | | 2.9 | 100 |
| Hematocrit, mean(sd) | 10.6 | | 9.18 | 100 |
| Platelet count(×10^9^/L), mean(sd) | 3.25 | | 2.56 | 100 |
| WBC^d^ (×10^9^/L), mean(sd) | 3.3 | | 2.49 | 14.55 |
| GRAN^e^ (%), mean(sd) | 4.89 | | 3.87 | 100 |
| TC^f^ (mmol/L), mean(sd) | 12.23 | | 13.03 | 100 |
| L-DLC^g^ (mmol/L), mean(sd) | 13.17 | | 9.85 | 100 |
| H-DLC^h^ (mmol/L), mean(sd) | 14.54 | | 11.01 | 21.35 |
| TG^i^ (mmol/L), mean(sd) | 9.22 | | 8.68 | 100 |
| K+^j^ (mmol/L), mean(sd) | 2.72 | | 2.41 | 100 |
| Serum bilirubin (μmol/L), mean(sd) | 12.43 | | 11.08 | 100 |
| LVEDd^k^(mm), mean(sd) | 24.68 | | 24.59 | 100 |
| LVEF^l^ (%), mean(sd) | 22.8 | | 22.88 | 100 |
| Malignant arrhythmia, n(%) | 0.96 | | 1.23 | 100 |
| CKMB^m^(IU/L), mean(sd) | 34.59 | | 32.94 | 100 |
| **Treatment, n(%)** |  | |  |  |
| Primary PCI^n^ | 1.16 | | 0.84 | 100 |
| Emergency CABG^o^ | 1.16 | | 0.84 | 100 |
| Thrombolysis | 1.16 | | 0.84 | 100 |
| Aspirin | 0.36 | | 0.88 | 100 |
| P2Y12 | 0.91 | | 1.53 | 100 |
| GPIIbIIIa | 3.34 | | 5.07 | 100 |
| Heparin | 2.78 | | 3.48 | 100 |
| Oral anticoagulant | 2.48 | | 3.87 | 100 |
| Statin | 6.29 | | 7.82 | 100 |
| Nitrate | 1.06 | | 2.14 | 100 |
| Betablocker | 0.98 | | 2.13 | 100 |
| Calcium channel blocker | 1.33 | | 2.56 | 100 |
| ACEI/ARB | 1.07 | | 2.29 | 100 |
| Antiarrhythmic drug | 1.21 | | 2.38 | 100 |
| Aldosterone antagonist | 1.06 | | 2.29 | 100 |
| Diuretics | 1.08 | | 2.24 | 100 |
| Non-statin lipid-lowering drug | 1.36 | | 2.67 | 100 |
| Acid-suppressing drug | 1.11 | | 2.32 | 100 |
| TCM^p^ | 1.35 | | 2.73 | 100 |
| **Medical History, n(%)** |  | |  |  |
| History of smoking | 0.43 | | 1.07 | 100 |
| History of drinking | 0.58 | | 1.13 | 100 |
| Dyslipidemia | 12.22 | | 15.13 | 100 |
| Diabetes | 3.76 | | 4.94 | 100 |
| Prior Angina | 8.57 | | 6.08 | 100 |
| Prior MI | 7.15 | | 4.56 | 100 |
| Prior CABG | 4.94 | | 1.76 | 100 |
| Prior Heart Failure | 6.19 | | 2.62 | 100 |
| Prior stroke | 4.97 | | 2.03 | 100 |
| Prior PVD | 5.4 | | 2.53 | 100 |
| Prior aortic disease | 5.45 | | 2.71 | 100 |
| Chronic Kidney Disease | 6.03 | | 3.21 | 100 |
| COPD^q^ | 5.68 | | 2.88 | 100 |
| History of Rheumatic diseases | 5.71 | | 2.4 | 100 |
| History of cancer | 5.5 | | 2.26 | 100 |
| History of stomach ulcer | 7.14 | | 2.83 | 100 |
| History of liver disease | 6.37 | | 2.64 | 100 |
| History of major bleeding | 7.11 | | 2.93 | 100 |
| **Living habit, n(%)** |  | |  |  |
| Current drinking | 0.58 | | 1.13 | 100 |
| Like-greasy | 44.2 | | 49.19 | 100 |
| Regular-Exersice | 30.56 | | 32.76 | 100 |
| Current smoking | 0.43 | | 1.07 | 100 |
| **Social economics, n(%)** |  | |  |  |
| Hospital-eastern | 0 | | 0 | 100 |
| Hospital equiped with CCU^r^ | 0 | | 0 | 100 |
| Hospital-capability of primary PCI | 0 | | 0 | 100 |
| Hospital-northern | 0 | | 0 | 100 |
| Hospital-capability of thrombolysis | 0 | | 0 | 100 |
| Hospitals annually admitting ≥100 patients with AMI^s^ | 0 | | 0 | 100 |
| Insurance(Self-paying) | 5.23 | | 4.3 | 100 |

^a^ **SBP:** systolic blood pressure; ^b^**EMS:** emergency medical service; ^c^**AWI:** anterior wall infarction; ^d^**WBC:** white blood cell; ^e^**GRAN:** neutrophilic granulocyte; ^f^**TC:** total cholesterol; ^g^**LDL-C:** low density lipoprotein-cholesterol; ^h^**HDL-C:** high density lipoprotein-cholesterol; ^i^**TG:** Triglyceride; ^j^**K+:** potassium; ^k^**LVEDd:** left ventricular end diastolic diameter; ^l^**LVEF:** left ventricular ejection fraction; ^m^**CKMB:** creative kinase MB; ^n^**PCI:** percutaneous coronary intervention; ^o^**CABG:** coronary artery bypass graft; ^p^**TCM:** traditional Chinese medicine; ^q^**COPD:** chronic obstructive pulmonary disease; ^r^**CCU:** coronary care unit; ^s^**AMI:** acute myocardial infarction;

##### Table S4 Hyper-parameters in the XGBoost model

| **Parameters** | **Type** | **Explanation** | **Values** |
| --- | --- | --- | --- |
| booster | string | Specify which booster to use: gbtree, gblinear or dart | ‘gbtree’ |
| max_depth | int | Maximum tree depth for base learners | 3 |
| n_estimators | int | Number of boosted trees to fit | 1000 |
| learning_rate | float | Boosting learning rate | 0.01 |
| gamma | float | Minimum loss reduction required to make a further partition on a leaf node of the tree. | 5 |
| min_child_weight | int | Minimum sum of instance weight (hessian) needed in a child. | 20 |
| scale_pos_weight | float | Balancing of positive and negative weights. | 0.8 |

##### Table S5 Hyper-parameters in the random forest model

| **Parameters** | **Type** | **Explanation** | **Values** |
| --- | --- | --- | --- |
| n_estimators | int | The number of trees in the forest | 1000 |
| max_depth | int | The maximum depth of the tree. | 3 |
